# Supplementary material for: Proteasomal Degradation of Mutant Huntingtin Exon1 Regulates Autophagy
Source: Cells. 2025 Dec 30;15(1):68. doi: 10.3390/cells15010068 (PMC12785617; doi:10.3390/cells15010068)
Supplement: Supplementary file 1 [file cells-15-00068-s001.zip › cells-4015257-supplementary.pdf]

Table S1. Yeast strains used in this study

**Strains**	**Genotype**	**Reference**
Y300	Mat**a** *ura3-1, his3-11,15 leu2-3,112 trp1-1, ade2-1, can1-100*	Lab Stock
3216-1-1	Mat**a** *pep4Δ::KanMX VPH1-mApple-Sphis5*$^{+}$ P_{GAL}*FLAG-Htt103QP-GFP-URA3*	Lab stock
3818-3-3	Mat**α** *ubr1::KanMX pep4Δ::NAT VPH1-mApple-Sphis5*$^{+}$ P_{GAL}*FLAG-Htt103QP-GFP-URA3*	This study
4082-1-2	Mat**a** *san1::KanMX pep4Δ::NAT VPH1-mApple-Sphis5*$^{+}$ P_{GAL}*FLAG-Htt103QP-GFP-URA3*	This study
3232-4-4	Mat**a** *cdc48-3 pep4Δ::KanMX VPH1-mApple-Sphis5*$^{+}$ P_{GAL}*FLAG-Htt103QP-GFP-URA3*	This study
3409-1-3	Mat**a** *ufd1-2 pep4Δ::Leu2 VPH1-mApple-Sphis5*$^{+}$ P_{GAL}*FLAG-Htt103QP-GFP-URA3*	Lab stock
3402-1-1	Mat**a** *npl4-1 pep4Δ::Leu2 VPH1-mApple-Sphis5*$^{+}$ P_{GAL}*FLAG-Htt103QP-GFP-URA3*	Lab stock
4077-1-1	Mat**α** *ubx1::KanMX pep4Δ::NAT VPH1-mApple-Sphis5*$^{+}$ P_{GAL}*FLAG-Htt103QP-GFP-URA3*	This study
4073-1-1	Mat**a** *ubx2::KanMX pep4Δ::NAT VPH1-mApple-Sphis5*$^{+}$ P_{GAL}*FLAG-Htt103QP-GFP-URA3*	This study
4075-1-1	Mat**α** *ubx3::KanMX pep4Δ::NAT VPH1-mApple-Sphis5*$^{+}$ P_{GAL}*FLAG-Htt103QP-GFP-URA3*	This study
3773-12-2	Mat**a** *ubx4::KanMX pep4Δ::NAT VPH1-mApple-Sphis5*$^{+}$ P_{GAL}*FLAG-Htt103QP-GFP-URA3*	This study
4074-13-1	Mat**a** *ubx5Δ::KanMX pep4Δ::NAT VPH1-mApple-Sphis5*$^{+}$ P_{GAL}*FLAG-103QP-GFP-URA3*	This study
3771-1-2	Mat**a** *ubx6Δ::KanMX pep4Δ::NAT VPH1-mApple-Sphis5*$^{+}$ P_{GAL}*FLAG-103QP-GFP-URA3*	This study
3774-10-4	Mat**a** *ubx7Δ::KanMX pep4Δ::NAT VPH1-mApple-Sphis5*$^{+}$ P_{GAL}*FLAG-103QP-GFP-URA3*	This study
